# Harmonic Distortion of Blood Pressure Waveform as a Measure of Arterial Stiffness

**DOI:** 10.3389/fbioe.2022.842754

**Published:** 2022-03-30

**Authors:** Nicholas Milkovich, Anastasia Gkousioudi, Francesca Seta, Béla Suki, Yanhang Zhang

**Affiliations:** ^1^ Department of Mechanical Engineering, Boston University, Boston, MA, United States; ^2^ Vascular Biology Section, Boston University School of Medicine, Boston, MA, United States; ^3^ Department of Biomedical Engineering, Boston University, Boston, MA, United States; ^4^ Division of Materials Science and Engineering, Boston University, Boston, MA, United States

**Keywords:** harmonic distortion, radio telemetry, tangent modulus, blood pressure waveform, pulse wave velocity

## Abstract

Aging and disease alter the composition and elastic properties of the aortic wall resulting in shape changes in blood pressure waveform (BPW). Here, we propose a new index, harmonic distortion (HD), to characterize BPW and its relationship with other *in vitro* and *in vivo* measures. Using a Fourier transform of the BPW, HD is calculated as the ratio of energy above the fundamental frequency to that at the fundamental frequency. Male mice fed either a normal diet (ND) or a high fat, high sucrose (HFHS) diet for 2–10 months were used to study BPWs in diet-induced metabolic syndrome. BPWs were recorded for 20 s hourly for 24 h, using radiotelemetry. Pulse wave velocity (PWV), an *in vivo* measure of arterial stiffness, was measured in the abdominal aorta *via* ultrasound sonography. Common carotid arteries were excised from a subset of mice to determine the tangent modulus using biaxial tension-inflation test. Over a 24-h period, both HD and systolic blood pressure (SBP) show a large variability, however HD linearly decreases with increasing SBP. HD is also linearly related to tangent modulus and PWV with slopes significantly different between the two diet groups. Overall, our study suggests that HD is sensitive to changes in blood pressure and arterial stiffness and has a potential to be used as a noninvasive measure of arterial stiffness in aging and disease.

## Introduction

Arterial stiffening is a significant contributor to the progression of cardiovascular diseases, including hypertension, diabetes mellitus, stroke, heart failure, and renal failure, which are the leading cause of mortality in the developed countries (Roman et al., 2000; Yambe et al., 2004; Rucka et al., 2015; O’Rourke et al., 2016). Arteries gradually stiffen with aging, which can lead to hypertension (HT) (Sun, 2015). Diabetic patients, however, show accelerated arterial stiffening with elevated blood pressure (BP) at a relatively young age compared to nondiabetic subjects (Chaturvedi, 2007; Loehr et al., 2016). BP is routinely used as a critical clinical measure for the diagnosis of HT. Additional parameters derived from BP and artery dimensions, such as distensibility and compliance, have also been widely used both in clinics and research as indicators of the mechanical properties of arteries (Glasser et al., 1997; Kannel et al., 2003; Bundy et al., 2017). However, these parameters solely rely on the systolic, mean, and diastolic pressure values, and the inherent biomechanical information associated with the shape of the BP waveform (BPW) is not considered. For example, there is a marked difference in BPW in central aortic pressure as well as pressure measured in upper limb arteries between young and older individuals due to wave reflection (Hirata et al., 2006). Arterial stiffening results in a pressure augmentation from the superposition of the propagating and reflected waves, which increases the peak of the waveform in the systolic phase of the cardiac cycle (van Varik et al., 2012). Therefore, the features of BPW are associated with arterial stiffness; however, the phenomenon is compounded by the nonlinear elastic behavior of the vascular wall and how pathologic changes in wall properties contribute to changes in BPW shape are not well understood.

The BPW is composed of a propagating wave, generated by cardiac contraction, and a reflected wave, from peripheral vessels to the proximal aorta. In clinical practice, indexes based on BPW are limited by their sole use of pressure differences (systolic minus diastolic) based on blood pressure measurements done on peripheral arteries (brachial arteries). One of the most widely used parameters, augmentation index (AI), quantifies the difference in pressure between the propagating wave and the reflected wave. Studies that sought to link AI to arterial stiffening and HT have shown that AI and pulse wave velocity (PWV), the gold standard clinical measure of arterial stiffness (Blacher et al., 1999; Nichols, 2005), are correlated with age, height, SBP, cholesterol and pulse pressure (Blacher et al., 1999; Brooks et al., 1999; Wilkinson et al., 2000; Brooks et al., 2001; Wilkinson et al., 2002; Nichols, 2005). Interestingly, AI and PWV were not strongly correlated with one another (Jerrard-Dunne et al., 2008). Females tend to have higher AI, an effect that seems to be independent from height and heart rate (Hayward and Kelly, 1997; Gatzka et al., 2001; Mitchell et al., 2010). This sex dependence of AI is present since prepuberty. Elevated AI is present in young girls (age 8.0 ± 0.1 years) compared to males of the same age, independent of factors that generally result in earlier wave reflection and increased AI, such as height or arterial stiffness (Ayer et al., 2010). Another BP index, reflection magnitude, is measured as the relative pressure magnitude of the reflected wave compared to the forward wave, which was suggested to better represent the overall changes due to the reflected wave (Mitchell, 2008; Mitchell et al., 2008; Mitchell et al., 2010). However, the correlation between reflection magnitude and AI is less than 0.6 (Segers et al., 2007a). Additionally, both fail to assess changes in the waveform beyond a simple increase in pressure, neglecting BPW features such as the time delay between the forward and reflected wave, which may also change the overall shape of the BPW (Ageenkova and Purygina, 2011; Theodor et al., 2014).

In this study, we aim to establish a relationship between BPW and arterial wall stiffness to understand the complex interplay between hemodynamic flow and the nonlinear mechanical properties of the arterial wall and how they manifest in BPW. To this end, we introduced a new index characterizing BPW, harmonic distortion (HD), which we hypothesize will provide a more comprehensive assessment of the BPW. A mouse model of diet-induced metabolic syndrome was used to validate the effectiveness and applicability of this new index. The BPW, collected using radiotelemetry, were analyzed for two diet groups. HD based on spectral analysis of the BPW was obtained and used to quantify the distortion of the BPW and compared to established stiffness indexes.

## Materials and Methods

### Animal Model

All procedures were approved by the Boston University Institutional Animal Care and Use Committee. Male (*n* = 29) C57Bl/6J mice were purchased from the Jackson Laboratory (Bar Harbor, ME, United States) at 7 weeks of age. After 1 week of acclimation, the 2 month old (-mo) mice were fed a control normal diet (ND: 4.5% fat, 0% sucrose, catalog number D09071702, Research Diets, New Brunswick, NJ, United States) or high fat, high sucrose diet (HFHS: 35.5% fat, 16.4% sucrose) ad libitum (catalog numbers D09071703, Research Diets, New Brunswick, NJ, United States). In this model of diet-induced metabolic syndrome, mice develop hyperinsulinemia, glucose-intolerance, increased arterial stiffness and hypertension within 8 months on diet, closely mimicking the human metabolic syndrome, as previously described (Weisbrod et al., 2013). Mice were kept in 12 h light/dark cycles in temperature- and humidity-controlled rooms. Radiotelemetry was surgically implanted to record BPWs continuously for 20 s on the hour for 24 h in conscious, freely moving mice. Mice were divided into subsets for various experimental conditions. One subset of mice (*n* = 6) had baseline BPW measurements taken at 2-months, before initiation of the HFHS diet, and subsequently fed the HFHS with measurements taken longitudinally at 3- and 4-months. A second subset of mice (ND: *n* = 5, HFHS: *n* = 8) underwent radiotelemetry implantation procedures at 6-months and had BPW measurements taken at 8- and 10-months. A third subset of mice were fed ND (*n* = 6) or HFHS (*n* = 4) and euthanized at 4- and 10-months for *in vitro* mechanical analysis of their right carotid arteries.

### *In vivo* Blood Pressure Measurements

**FIGURE 1:**
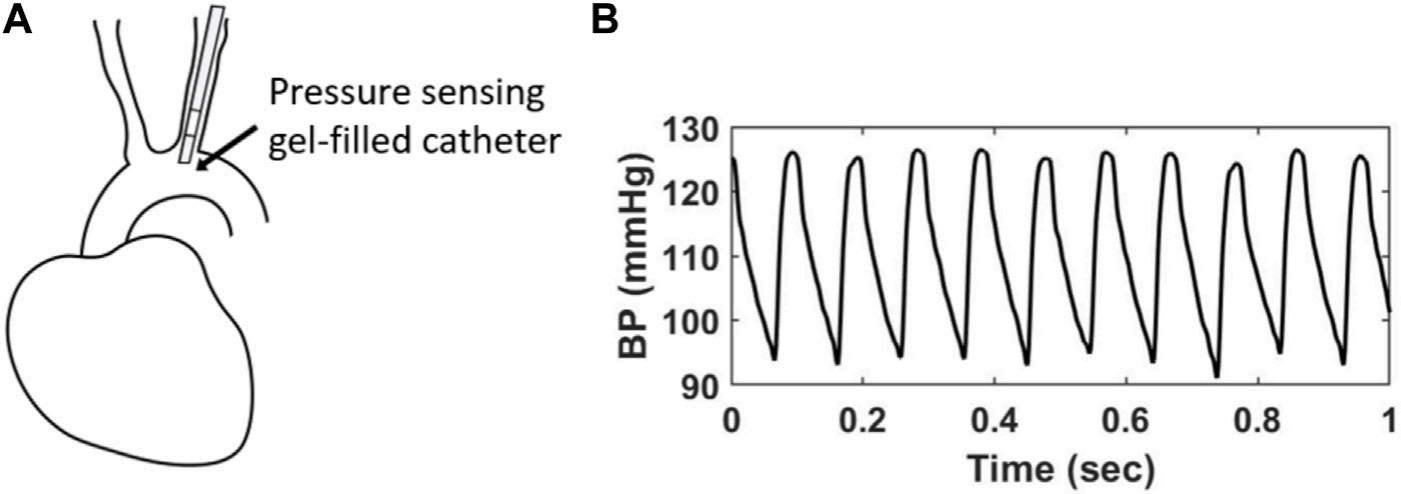
**(A)** A gel-filled pressure catheter is surgically inserted in the left common carotid artery of a mouse, with the pressure-sensing region (4 mm) resting in the aortic arch. **(B)** Example of *in vivo* BPWs over 1 s time period measured using radiotelemetry.

The BPW was measured using radiotelemetry (Data Sciences International, St Paul, MI, United States) that was implanted following standard surgical procedures, as we previously described (Weisbrod et al., 2013). Briefly, mice were kept anesthetized (1–2% isofluorane) on a heating pad, while a gel-filled pressure catheter was carefully inserted in the aortic arch via catheterization of the left carotid artery (Figure 1A). Therefore, all BPW measurements made with radiotelemetry are local to the aortic arch and indicative of central pressure. After recovery from surgery (1–2 weeks), BPW recordings were acquired for 20 s hourly over a 24-h period in the conscious, freely moving mice. BPWs are collected wirelessly through an external receiver and a data processing matrix (Figure 1B). This resulted in a total of 24 distinct recordings for each mouse, with each recording consisting of many individual waveforms (Figure 1B). The SBP values from each waveform were then collected and separated into 16 blood pressure bins, ranging from 80–180 mmHg, for clarity of group overlap. Normalized probability density distributions of the average SBP of each bin was then generated for every mouse. The distributions were then fitted with Gaussian functions using a MATLAB program.

### Harmonic Distortion

**FIGURE 2:**
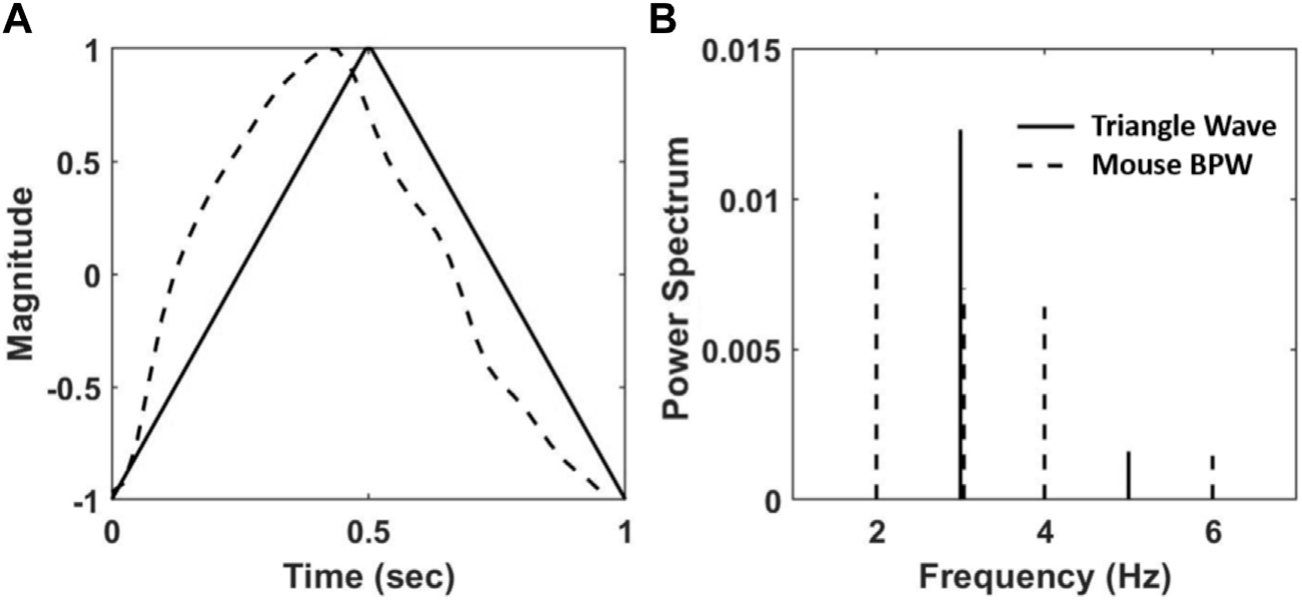
**(A)** A triangular wave (solid line) and a normalized BPW from radiotelemetry (dashed line). **(B)** The normalized power spectrum, calculated as the squared fraction of Fourier coefficients at integer multiple of the fundamental frequency of the waveforms in **(A)** at 1 Hz.

To quantitatively compare BPWs, we introduce an index, harmonic distortion (HD), which will be used to quantify the shape change of BPW. HD is defined based on the discrete Fourier transform (DFT) of the BPW, as:

$$HD=\frac{\sum_{k=2}^{6}{\left|A_{k}\right|}^{2}}{{\left|A_{1}\right|}^{2}}$$ (1)

where ${\left|A_{k}\right|}^{2}$ are the Fourier coefficients of a single BPW multiplied by their complex conjugates. Thus, HD is essentially the ratio of energy above the fundamental frequency to that at the fundamental frequency of the waveform. Here, Fourier coefficients higher than the sixth were assumed to be negligible since they did not significantly contribute to the HD value. For an ideal sinusoidal wave, the HD value is 0. As an illustration, the normalized power spectrum, $\frac{{\left|A_{k}\right|}^{2}}{{\left|A_{1}\right|}^{2}}$ , were obtained for a triangle wave and a mouse BPW (Figure 2A) using a custom MATLAB code according to Eq. 1 and plotted in Figure 2B. The triangle wave has a HD value of 0.014 whereas the sample BPW waveform is more distorted with an HD value of 0.025.

For HD analysis of the BPW, waveforms corresponding to a single heartbeat were isolated as they appear in Figure 2A using custom MATLAB code by identifying the diastolic BPs. Eq. 1 was then used to determine the HD value for each individual waveform. This analysis was performed on all mice and for all 24 BPW recordings. The corresponding SBP, the maximum value of BP in an isolated individual waveform, was also recorded and 12 evenly spaced BP bins were created between the minimum and maximum SBP values. The bin size was chosen by studying multiple bin sizes and assessing their effect on the slope of the regression line. Because the bins are weighted, the slope of these regressions is not sensitive to bin sizes (Supplementary Figure S1). Hence, the bin size was kept consistent for all mice. The mean and standard deviation (SD) of HD corresponding to each BP bin were calculated. HD values that were two SD outside of the mean HD value were removed. The mean and SD of HD were then recalculated for each BP bin as well as the corresponding mean BP, resulting in 12 data points for each mouse. A weighted linear regression between the mean HD and the mean BP was performed for each age and diet group. The weight of each data point was determined by the ratio of the number of HD values in the BP bin to the total number of HD values recorded for the mouse.

### Pulse Wave Velocity Measurements

Blood flow waves at two locations along the abdominal aorta, one proximal and one distal to the heart, using the renal artery as anatomical reference, were obtained in each age and diet group using high-resolution Doppler ultrasound (Vevo2100, Fujifilm-Visualsonics, Toronto, ON, Canada), as we previously described (Weisbrod et al., 2013). PWV, the rate at which BP waves travel along the aorta, was calculated as the ratio of the distance between the two locations and the difference in arrival times of two consecutive flow waves, using the foot-to-foot method and the ECG as fiducial point, over 5–10 cardiac cycles for each mouse.

### *In vitro* Determination of Tangent Modulus

To establish the relationship between HD and arterial stiffness, tangent modulus of the common carotid arteries was obtained from a subset of mice from the 4-months ND (*n* = 3), 4-months HFHS (*n* = 2), 10-months ND (*n* = 3) and 10-months HFHS (*n* = 2) groups. Biomechanical characterization of these arteries was performed in a separate study by Gkousioudi et al. (2022), from which the circumferential Cauchy stress-stretch responses were obtained from biaxial extension-inflation tests, and then fitted using a four-fiber family constitutive model with the following strain energy function (Ferruzzi et al., 2013; Ferruzzi et al., 2018a):

$$W\left(C,a^{i}\right)=\frac{c}{2}\left(I_{C}-3\right)+\overset{4}{\underset{i=1}{\sum }}\frac{c_{1}^{i}}{{\mathrm{4c}}_{2}^{i}}\left\{exp\left[c_{2}^{i}{\left(I_{4}^{i}-1\right)}^{2}\right]-1\right\}$$ (2)

In this constitutive model, arterial wall is considered a composite of the isotropic extracellular matrix (elastic fibers, cells and ground substance) and anisotropic collagen fibers, which are assumed to be oriented in four directions: axial ( $\alpha^{1}$ = 0^o^), circumferential ( $\alpha^{2}$ = 90^o^), and diagonal $\left(\alpha^{3}=-\alpha^{4}=\alpha \right)$ . c and $c_{1,2}^{i}$ are material parameters for the isotropic matrix and the collagen fibers, respectively. $C=F^{T}F$ is the Cauchy-Green deformation tensor with ***F*** being the deformation gradient. $I_{C}=trC$ is the first invariant of ***C***. $I_{4}^{i}=a^{i}\cdot Ca^{i}$ represents the invariant that is associated with the $i^{th}$ fiber family, and $a^{i}$ is the unit vector that denotes the orientation of the $i^{th}$ fiber family.

In this study, circumferential tangent modulus, *C*_*θθθθ*_, was calculated as the first derivative of the Cauchy stress-stretch expressions using the best-fitted model parameter values as (Baek et al., 2007; Gkousioudi et al., 2022):

$$C_{\theta \theta \theta \theta }=2t_{\theta \theta }+4\lambda_{\theta }^{4}\left(\frac{\partial^{2}W}{\partial \lambda_{\theta }^{2}\partial \lambda_{\theta }^{2}}+2{\text{sin}}^{4}\alpha \frac{\partial^{2}W}{\partial \lambda_{\theta }^{2}\partial \lambda_{\theta }^{2}}\right)$$ (3)

where $t_{\theta \theta }$ is the Cauchy stress in the circumferential direction, $\lambda_{\theta }$ is the circumferential stretch, W is the strain energy function based on the four-fiber family constitutive model (Eq. 2), and *α* is the orientation angle of the diagonal collagen fiber families with respect to the longitudinal direction estimated during nonlinear regression model fitting (Ferruzzi et al., 2013; Ferruzzi et al., 2018a; Gkousioudi et al., 2022). Tangent modulus, $C_{\theta \theta \theta \theta }$ , was then calculated as a function of the circumferential stretch $\lambda_{\theta }$ .

To establish a proper relationship between the HD obtained from *in vivo* measurements and the tangent modulus from the *in vitro* measurements, perivascular pressure, the pressure difference between the luminal and transmural pressures (Goshy et al., 1979; Kim et al., 2013; Ferruzzi et al., 2018b), needs to be considered to obtain the *in vivo* transmural pressure. Here, an estimation of average perivascular pressure 13.22 mmHg was obtained by incrementally adjusting its value for the best linear regression fit between HD and tangent modulus for each mouse. This average perivascular pressure value was then subtracted from the *in vivo* luminal SBP of each mouse to obtain transmural SBP.

### Statistical Analysis

Wilcoxon rank sum tests were performed on the low and high SBP means of each mouse group after normality was not found using one-sample Kolmogorov-Smirnov tests. HD values for each mouse group were found to be not normally distributed using paired Kolmogorov-Smirnov tests. Therefore, Friedman tests were performed on HD values between mouse groups when groups had the same number of subjects and Skillings-Mack tests were performed when comparing groups with a different number of subjects. A *p*-value of 0.05 or lower was considered statistically significant.

## Results

### Blood Pressure Variability

**FIGURE 3:**
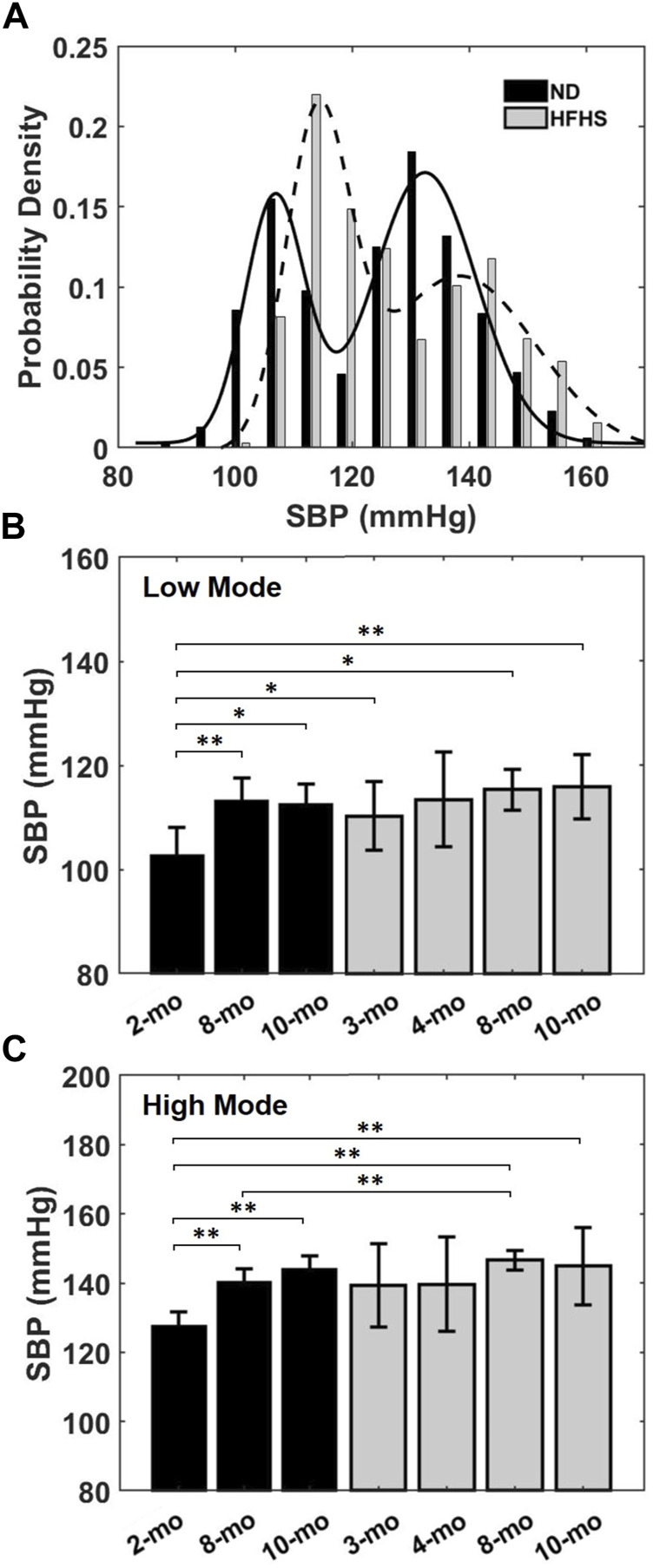
**(A)** Representative bimodal probability density distribution of SBP over a 24-h period for a 2-months ND and a 3-months HFHS mouse. Solid and dashed lines are Gaussian distributions fit to the data. The low and high pressure values of the bimodal SBP distribution for each age and diet groups were averaged and showed in **(B,C)** for the low and high pressure means, respectively, for the age and diet groups (^*^ *p* < 0.05; ^**^ *p* < 0.01).

BPW data were firstly used to assess the variation of BP over a 24-h period. Probability density distributions of the SBP, i.e., the peak of the individual BPWs, were plotted for each mouse. Figure 3A shows a typical bimodal distribution of the SBP over a 24-h period. The data were fit with the sum of two Gaussian distributions with each peak centering at a low and high systolic blood pressure. The low- and high-pressure values at the two peaks were averaged for each age and diet group. The SBP distribution of the HFHS mouse showed a wider range of high SBP compared to the ND mouse with the two peaks correlating with age and diet. There was a significant increase in the SBP means with age in the ND groups (Figures 3B,C), however this trend was absent in the HFHS groups. The 2-months ND group’s mean low- and high-pressure values are statistically lower than all other groups, except the 4-months HFHS and the 3-months HFHS high pressure mean.

In the current study, 5 of the 19 analyzed BPW were found to either have one or three modes (*n* = 2 and 3, respectively). The single mode’s mean corresponded to the group high pressure average and was included as such. In the three modes case, a mean value of either high or low pressure was only included if the Gaussian distribution could be fitted to the pressure distribution, and the mode with a mean outside of one standard deviation of the low or high mean was discarded.

### Harmonic Distortion

**FIGURE 4:**
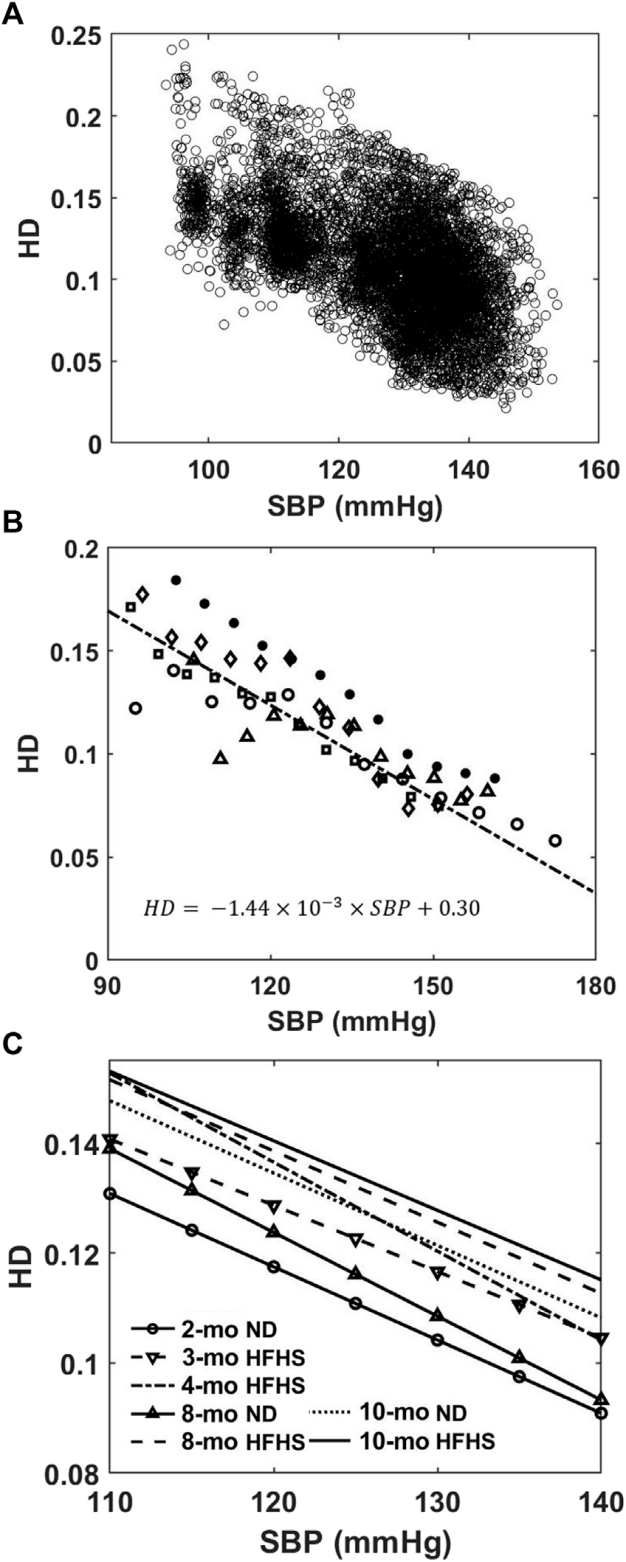
**(A)** HD values of individual BPW are plotted as a function of the corresponding wave’s SBP. **(B)** Mean HD values within evenly spaced BP bins for the 8-months ND group (*n* = 5). Different symbols represent different mice in the group. The solid line represents a weighted linear regression (*r* ^2^ = 0.78) to the data from the group. **(C)** Linear regressions between HD and luminal SBP for the 7 age and diet groups. The linear relationships consistently trend upward with increasing age and HFHS diet.

HD analysis showed that the HD values from the isolated individual BPW were inversely related to SBP over a 24-h period, as shown in Figure 4A for an 8-months ND mouse. The HD values range from ∼0.24 to 0.03 between SBP of 93 and 153 mmHg. To study the trend between HD and SBP, evenly spaced blood pressure bins were used to find a mean HD within each bin. This mean HD is then plotted with the mean SBP of that bin for every mouse. Despite the large spread of HD seen in Figure 4A, following binning, a linear relation was apparent as shown for the 8-months ND group in Figure 4B.

Linear regression curves were then generated in a similar manner for all groups and plotted in Figure 4C. An upward vertical shift in the curves is observed with age and diet, and the shift becomes more pronounced for older mice and mice on the HFHS diet (Figure 4C). In the pressure range of 110–140 mmHg SBP that all mice experience, the Friedman test, which evaluated the difference between groups’ HD values, reveals the relative effect of the age on ND or HFHS diet (Table 1). The tests paired each of the seven age and diet groups with one another, using the rising SBP readings as repeated measures and the corresponding HD values as dependent variables. Many of these pairings were found to be highly significant with *p* < 0.001, suggesting a significant difference in HD between the age and diet groups. However, no significant difference was found the between 2-months ND and 3-months HFHS (*p* = 0.375), 8-months ND and 4-months HFHS (*p* = 0.977), 8-months HFHS and 10-months HFHS (*p* = 0.549), and the two 10-months groups (*p* = 0.346).

**TABLE 1:** The *p*-values were obtained from Friedman test and Skillings-Mack test between grops. 2-months ND (*n* = 6), 3-months HFHS (*n* = 6), all other groups (*n* = 5). Tests were performed on average HD values with SBP bins ranging from 110–140 mmHg between each age/diet group.

	3-months HFHS	4-months HFHS	8-months ND	8-months HFHS	10-months ND	10-months HFHS
2-months ND	0.375	<0.001	<0.001	<0.001	<0.001	<0.001
3-months HFHS		<0.001	<0.001	<0.001	<0.001	<0.001
4-months HFHS			0.977	0.002	0.007	0.002
8-months ND				<0.001	0.008	0.002
8-months HFHS					<0.001	0.549
10-months ND						0.346

### HD vs. Arterial Stiffness

**FIGURE 5:**
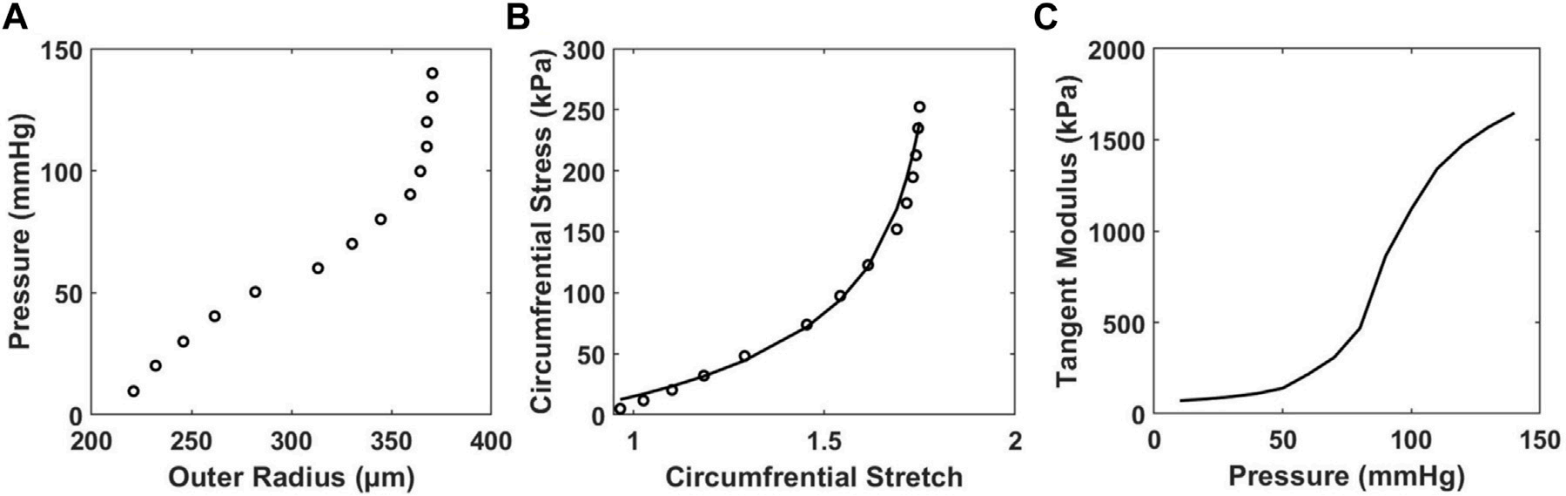
Representative results of **(A)** *in vitro* pressure-outer radius curves from biaxial extension-inflation test; **(B)** circumferential stress-stretch curves; and **(C)** tangent modulus vs. pressure of a carotid artery sample of a 4-months ND mouse with model parameter c = 20.540 kPa, $c_{1}^{1}$ = 3.742 kPa; $c_{2}^{1}$ = 0.030; $c_{1}^{2}\;$ = 11.267 kPa; $c_{2}^{2}$ = 0.043; $c_{1}^{3,4}$ = 0.008 kPa; $c_{2}^{3,4}$ = 1.373 kPa, and *α* = 46.352° (Gkousioudi et al., 2022).

To study the relationship between HD and arterial stiffness, we obtained tangent modulus measurements by first using the inflation test of carotid artery samples to obtain pressure-radius curves (Figure 5A), from which the circumferential stress-stretch relationships can be calculated (Figure 5B). At transmural pressures between 0–60 mmHg, the tangent modulus is nearly the same for each mouse, regardless of age and diet (Supplementary Figure S2). As pressure reaches about 60–70 mmHg, the tangent modulus increases prominently with pressure (Figure 5C, Supplementary Figure S2). Mice from the 10-months group were found to have a decrease in tangent modulus when compared with the 4-months group, which was accompanied by compromised energy storage capability and lower wall stress, as shown previously from others and our recent work (Gkousioudi et al., 2022). Mice on the HFHS diet appear to have lower tangent modulus values in the 4-months group, however this trend disappears in the 10-months group.

**FIGURE 6:**
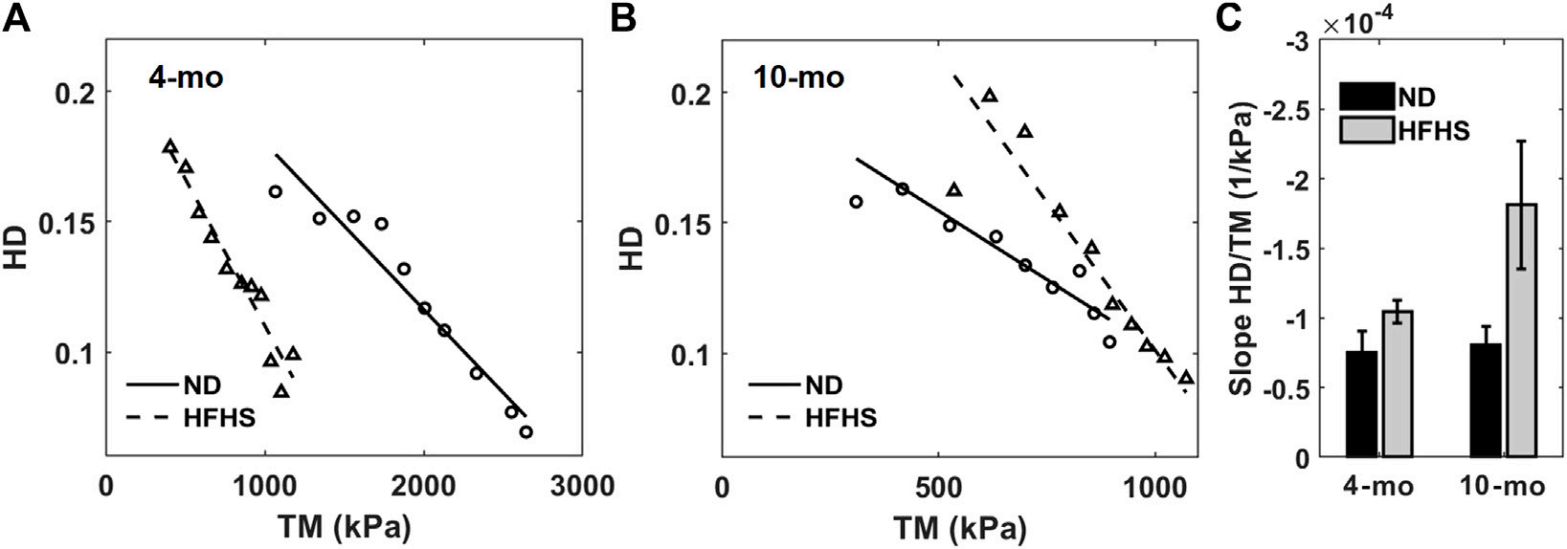
Harmonic distortion from *in vivo* spectral analysis as function of tangent modulus measured by *in vitro* biomechanical testing for the 4-months **(A)** and 10-months mice **(B)**, normal (circles and solid line) and HFHS diet (triangles and dashed line). Linear regressions from each group are also displayed. **(C)** Average slopes of the harmonic distortion vs. tangent modulus linear fitting for the age and diet groups.

For each mouse the HD value and tangent modulus at the same transmural pressure were plotted for the 4- and 10-months groups (Figures 6A,B) and fitted with a linear line. The average slopes of these linear fits are displayed in Figure 6C. Our results demonstrate there is a linear relationship between HD and tangent modulus obtained from *in vitro* biomechanical testing. This relationship is consistent within all mice (*n* = 10) from both ND and HFHS groups. Furthermore, the linear trend appears to be steeper with HFHS diet. Average perivascular pressure was used in this study; however, it is not known whether perivascular pressure changes with aging and the development of metabolic syndrome. Adopting perivascular pressures at 13.22 ± 5 mmHg had an unnoticeable effect on the linear regressions between tangent modulus and HD (Supplementary Figure S2; Supplementary Table S1), although the effect of perivascular pressure on the slope of the linear regression needs to be further investigated.

**FIGURE 7:**
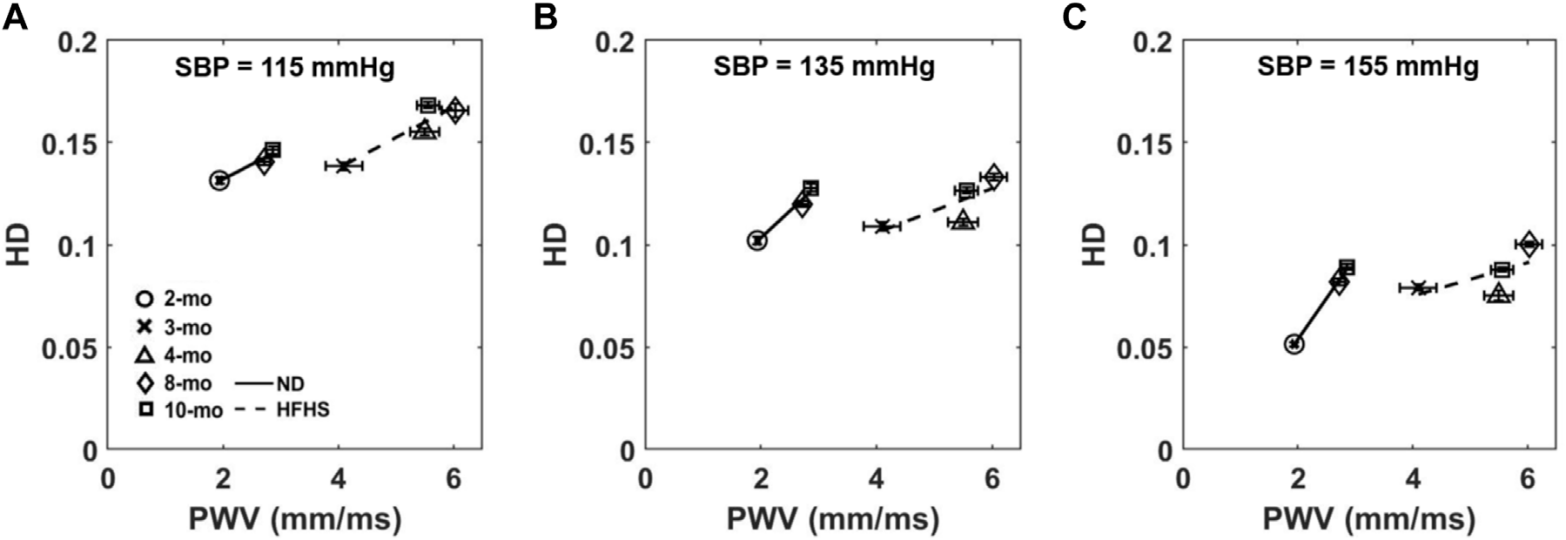
Average HD at 115 mmHg **(A)**, 135 mmHg **(B)**, and 155 mmHg **(C)** is plotted as a function of the group’s corresponding average PWV, collected *via* Doppler ultrasound, for each age and diet group.

The relationship between HD and PWV, a measure of arterial stiffness *in vivo*, was examined by plotting the mean HD values at 115, 135, and 155 mmHg against the average PWV values for each age and diet group (Figure 7). The slopes of the linear fits change at different pressures as well as the slope of the two diet groups. HD increases linearly with PWV for both the control ND group and the HFHS diet group with *R* ^2^ values between 0.375 and 0.998. The slopes of the linear relationship for the ND group increase with pressure with slopes being 0.015, 0.026, and 0.040 at 115, 135, and 155 mmHg, respectively. However, the plots for the HFHS groups decrease slightly with slopes being 0.015, 0.011, and 0.008 at 115, 135, and 155 mmHg, respectively.

## Discussion

BPW contains information on the coupled interactions among the forward propagating wave, reflected wave, as well as the arterial wall mechanics (Hirata et al., 2006; Ageenkova and Purygina, 2011; van Varik et al., 2012; Theodor et al., 2014). In this study a novel index, HD, was derived from the BPW that was measured *in vivo* using radio telemetry in mice. The nonlinear nature of the wall mechanics combined with the reflected wave alter the BPW by changing the time delay between the forward traveling and reflected waves (Amin et al., 2012) as well as the magnitude of these waves (Ageenkova and Purygina, 2011; Theodor et al., 2014). We hypothesized that HD would be sensitive to such changes. To test this, we studied the relationship between HD and other arterial stiffness measures obtained both *in vitro* and *in vivo*. Our results suggested that HD shows promise in assessing arterial stiffness as demonstrated by the linear correlation between HD and tangent modulus, an *in vitro* measure of arterial stiffness, as well as with PWV, an *in vivo* index of arterial stiffness. Furthermore, the slopes of the relationships show dependence on diet.

### Blood Pressure Variability

Both SBP and HD show variability within individual subjects. The BPW has beat-to-beat variability with SBP varying considerably based on many factors including: PWV, age, 24-h activity level, as well as brain and nerve function (Schillaci et al., 2012; Miao and Su, 2002; Stauss et al., 2008; Yoshimoto et al., 2011). From our results over the course of 24 h, SBP also varies largely with relative day-and-night activity cycles (Figure 3A). The distributions of SBP followed a bimodal Gaussian form, containing a low mean and high mean SBP in mice fed ND and HFHS diet (Figure 3), which likely corresponds with the day-and-night cycle in which subjects experience variable blood pressures related to their relative activity levels (Vliet et al., 2003; Van Vliet et al., 2006). The SBP distributions (Figure 3) indicate that diet-induced metabolic syndrome promotes a greater probability of high SBP. We have noted a significant increase in the low-pressure mode after just 1 month of HFHS diet (*p* = 0.0303). This suggests that the SBP distribution may contain an earlier manifestation of hypertension than mean BP, as used in a previous study (Weisbrod et al., 2013). High blood pressure is highly associated with increased risk of cardiovascular diseases (Kannel, 2000). Therefore, analysis of BP distribution and variability may provide insight on the physiological changes in cardiovascular function. Despite the variability in SBP and HD, our study shows that HD is linearly related to blood pressure; as SBP increases the corresponding isolated wave’s HD decreases (Figure 4A). The trend is measurable and consistent within each age and diet group (Figure 4C). Elevated blood pressure is a key measure of hypertension and cardiovascular risk, though such elevation is only observed at longer time scales, in the order of months. From our study, it is important to note that beat-to-beat variability exists in SBP and BPWs. Due to the relative activity level, the mice experience high SBP in the young groups (Figure 3A) as well as low SBP in the old group. The relationship between HD vs. SBP was established based on the beat-to-beat variability using 20’s BPW data. Such short-term fluctuations in SBP and BPW (Figure 3A and Figure 4A) and their relationships (Figure 4C) have demonstrated to be different between the age and diet groups. Additionally, a key finding of our study is that HD is strongly associated with arterial wall stiffness, an independent cardiovascular risk factor that cannot be derived from SBP alone.

### Harmonic Distortion Index

HD analysis of the BPW indicates that the BPW undergoes changes with age and diet-induced metabolic syndrome, both of which are linked to changes in arterial wall stiffness (Blacher et al., 1999; Nichols, 2005; Wilkinson et al., 2002; Brooks et al., 2001; Brooks et al., 1999; Wilkinson et al., 2000). The inverse linear relationship between HD and SBP (Figure 4B) suggests that HD is sensitive to changes in arterial stiffness, since with higher mean blood pressure, arterial stiffness increases (Figure 5C). This is further confirmed by the inverse linear relationship between HD and the tangent modulus (Figures 6A,B). At higher blood pressures, there is an increase in arterial stiffness due to the gradual recruitment of collagen fibers in the adventitia (Chow et al., 2014), which would impact the BPW and thus HD. Moreover, with arterial stiffening, the waveform peak is augmented due to the overlapping of the forward and reflected waves (van Varik et al., 2012), leading to higher distortion of the BPW (Figure 4C). This overlap is largely originated from increased speed of both wavelets, coupled with earlier reflections during arterial remodeling (van Varik et al., 2012). Spectral decomposition of stress waveforms *via* a Fourier transform has been used in a previous study to characterize mechanical nonlinearity of isolated rat aorta (Imsirovic et al., 2018). Future study is required to understand the role of nonlinear arterial wall properties in relation to HD.

The relationship between HD and arterial stiffness is liable to shift with age and, more significantly, diet. There is also an apparent increase in slope of this relation for each age and diet group (Figure 6C). Consistent with previous findings that hypertension develops after 6 months of HFHS diet (Weisbrod et al., 2013), the 8-months HFHS group has significantly higher HD values than the 8-months ND group (*p* < 0.001) (Table 1). Further, the two 10-months groups (both ND and HFHS) are not different with regards to their HD trends, which implies that changes due to diet have slowed or subsided by 10-months. Nonetheless, the two old ND groups (8-months and 10-months) contain significantly different HD values (*p* = 0.008) suggesting change in HD still occurs in ND aging after the mice have reached maturity. In contrast, the 8-months HFHS group’s HD values are not significantly different from 10-months HFHS (*p* = 0.549). This supports the idea that changes in the arterial wall mechanics have already occurred in the 8-months HFHS group. The efficacy of detecting this change supports HD as a noninvasive predictor of hypertension which warrants further studies.

### HD vs. Pulse Wave Velocity

PWV is a broadly used *in vivo* index of arterial stiffness that measures the velocity of the forward propagating arterial pressure wave (Blacher et al., 1999; Nichols, 2005). When studying the relationship between HD and PWV (Figure 7), it is important to note that HD, derived from beat-to-beat BPW, is pressure dependent while PWV is not. We found that HD and PWV to be linearly related but with a diminishing R squared value in the HFHS group related to increasing pressure (Figure 7). Further study is needed to better understand the pressure and diet dependency of this relationship. The positive HD-PWV relation is in contrast to that of the tangent modulus and HD, which demonstrated a negative relation (Figure 6). While the explanation for this is not entirely clear, these results suggest that the tangent modulus is also negatively related to PWV. Possible explanations include the following. The PWV measurements were made in the abdominal aorta while HD was obtained from BPWs at the aortic arch. Abdominal aorta has been reported to be more severely affected by vascular remodeling (Hayashi et al., 2010), which could account for the more dramatic change seen in PWV with age and diet as compared to HD (Figure 7). Notably, the ND and HFHS diet groups follow their own linear paths (Figure 4C). It is also important to point out that HD is derived from local BP measurement, taken in a point within the aortic arch where the radiotelemetry is surgically implanted within the mouse, while PWV is usually obtained over a segment length of ∼1 cm. This suggests that HD can potentially be used to reveal the regional dependent condition of the arterial wall, independently of PWV.

When considering HD, it is important to mention arterial impedance as both HD and impedance rely on Fourier transforms of the BPWs (Kelly and Fitchett, 1992). Impedance is calculated by the ratio of Fourier amplitudes from the BPW and velocity waveforms, which provides an impedance modulus and phase over a range of frequencies (Kelly and Fitchett, 1992), and the modulus has been shown to increase with age-related arterial stiffening (Reddy et al., 2003). However, impedance analysis requires invasive methods and it does not consider the effect of BP on Fourier coefficients from BPWs. Previous studies on input impedance have shown inconsistencies when relating impedance directly to PWV after adjusting for factors like mean blood pressure (Mitchell et al., 2002; Segers et al., 2007b). Our results of the HD dependence on SBP (Figure 4A) suggest that HD, or the Fourier coefficients, of BPWs change with SBP and such changes should be considered when studying BPW.

## Limitations

Our study has several limitations. Future studies including the 3 and 4 months ND groups may be necessary for direct comparison with the 3 and 4 months HFHS groups, although our previous study showed that the 2, 3, and 4 months ND groups do not show significant differences in arterial stiffness and blood pressure measures (Weisbored et al., 2013). Only male mice were used in this study. Dependence of BPW and HD index on sex also warrants further studies. Additionally, the number of mice should be increased to make quantitative comparisons between HD and tangent modulus among different age and diet groups. The radiotelemetry BPW readings were taken in the aortic arch at the left carotid artery. Due to the proximity of aortic arch and carotid arteries, it was assumed that pressure taken at the arch would be an acceptable estimation of pressure within the carotid artery in order to compare HD derived from BPWs with tangent modulus from direct *in vitro* stiffness. This study uses surgically implanted telemetry for BPW, but other more accessible approaches such as BP tonometry could produce the waveforms required for this analysis (Parry Fung et al., 2004; Feng and DiPetrillo, 2009). PWV was measured over the abdominal aorta. Differences between the material properties and the progression of arterial stiffening varies between arteries (Hayashi et al., 2010) and therefore regional variation in mechanical properties of arteries should be kept in mind while making comparisons. The interference between radiotelemetry and BPW is not known. The HD index cannot differentiate between reflection and local wall properties. Clinical studies are needed to dissect the contribution of individual factors, such as hemodynamics and wall properties, to BPW and HD.

## Conclusion

BPW contains a wealth of information from the interactions of the forward and backward propagating waves, arterial wall mechanics, and hemodynamics. In this study, HD is proposed as a novel index to assess changes in arterial mechanical function. We showed that HD, obtained based on Fourier transform of individual BPWs, is related to SBP, and other existing *in vitro* and *in vivo* arterial stiffness measures. HD is also sensitive to age and metabolic syndrome-induced changes in BPW. Instruments used to record BPWs are more readily available clinically than instruments used to record flow. Hence, our results demonstrate that HD has the potential to be used as a noninvasive and easily accessible means to assess cardiovascular risk in future clinical settings.

## Data Availability

The original contributions presented in the study are included in the article/Supplementary Material, further inquiries can be directed to the corresponding author.
