# Supplementary material for: Harmonic Distortion of Blood Pressure Waveform as a Measure of Arterial Stiffness
Source: Front Bioeng Biotechnol. 2022 Mar 30;10:842754. doi: 10.3389/fbioe.2022.842754 (PMC9006055; doi:10.3389/fbioe.2022.842754)
Supplement: Supplementary file 1 [file Presentation1.PDF]

(Supplemental Material)

Harmonic Distortion of Blood Pressure Waveform as a Measure of Arterial Stiffness

Nicholas Milkovich[1], Anastasia Gkousioudi[1], Francesca Seta[2], Bela Suki[3], and Yanhang Zhang[1,3,4]

[1]Department of Mechanical Engineering, Boston University, Boston, MA
[2]Vascular Biology Section, Boston University School of Medicine, Boston, MA
[3]Department of Biomedical Engineering, Boston University, Boston, MA
[4]Division of Materials Science & Engineering, Boston University, Boston, MA

Corresponding author:

Yanhang Zhang
Department of Mechanical Engineering
Department of Biomedical Engineering
Division of Materials Science & Engineering
Boston University
110 Cummington Mall
Boston, MA 02215
Phone: (617) 358-4406; Fax: (617) 353-5866
Email: yanhang@bu.edu

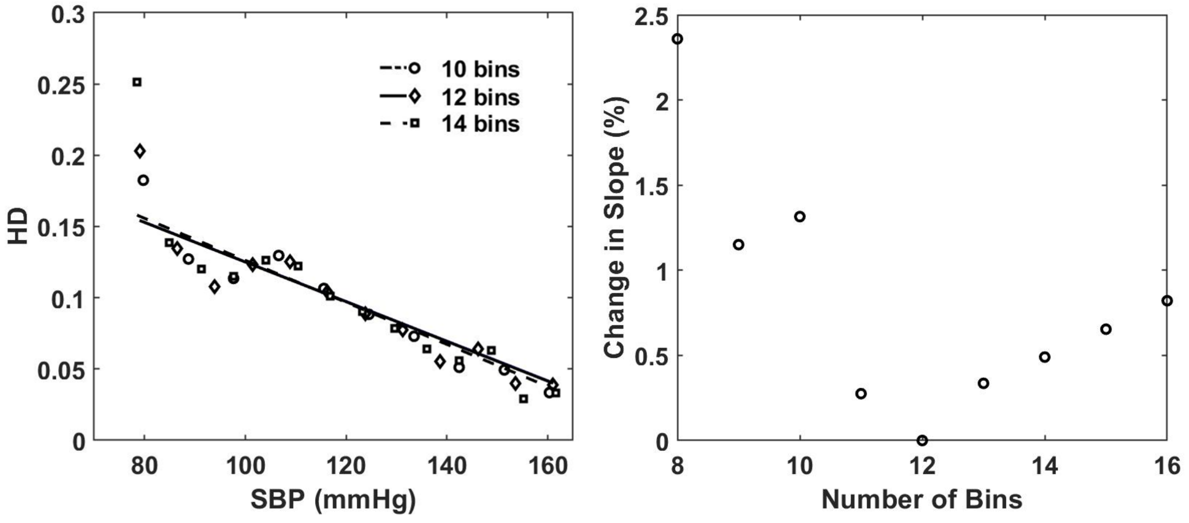

(a) (b)

Figure S1: (a) Weighted linear regressions of HD-SBP data from a 2-mo ND mouse after binning raw data points into 10, 12, and 14 bins. (b) Percent change in slopes of the 2-mo ND group using various SBP bin sizes to perform HD-SBP linear regressions. The percentage was calculated with respect to the slope using 12 bins.

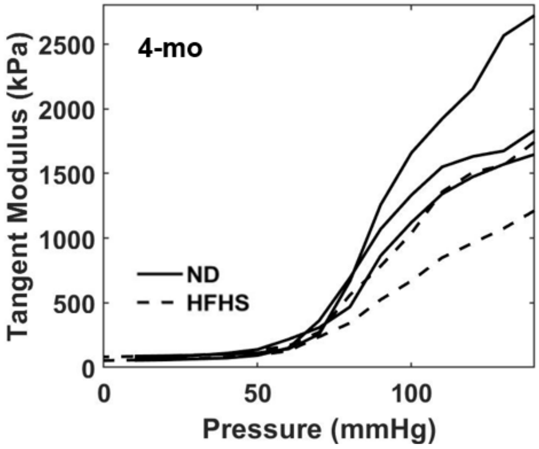

(a)

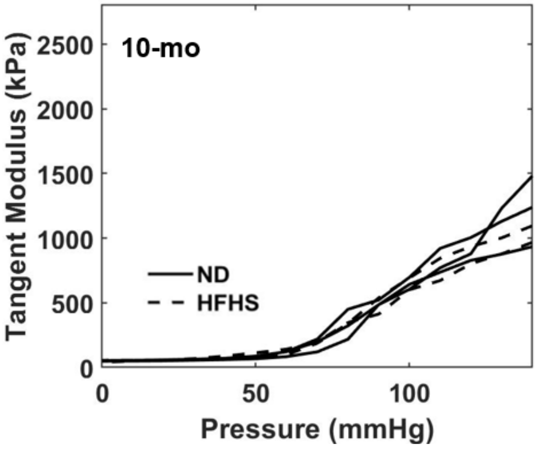

(b)

Figure S2: Tangent modulus calculated for the carotid artery samples from the 4-mo (a) and 10-mo (b) ND and HFHS groups at incremental transmural pressures ranging from 0 to 140 mmHg.

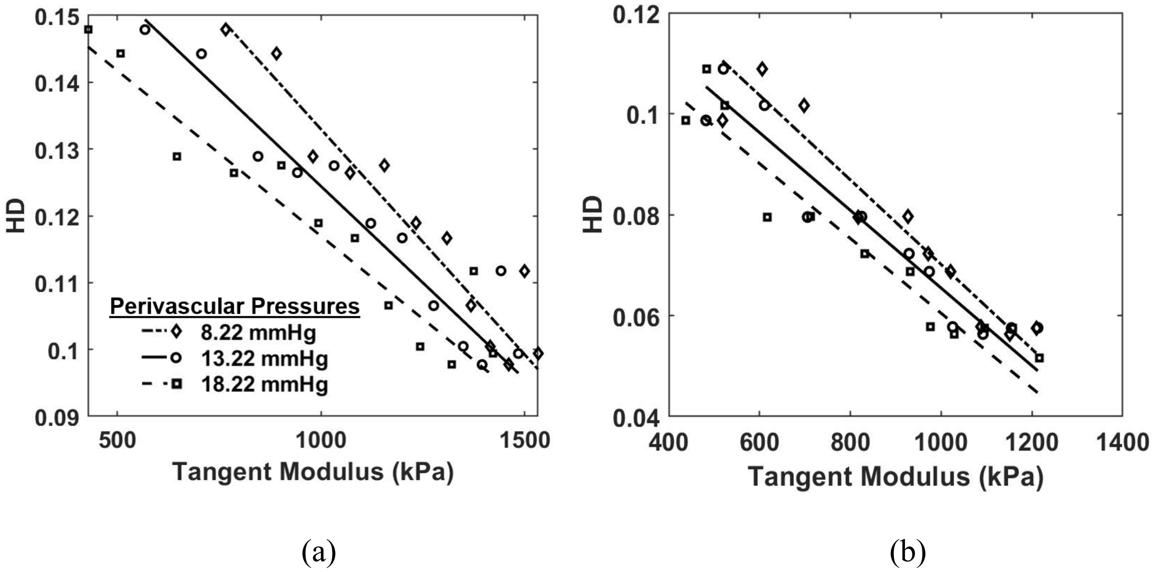

(a) (b)

Figure S3: Examples of linear regressions of HD-tangent modulus data from a 4-mo ND mouse (a) and 10-mo ND mouse (b) at 13.22 ± 5 mmHg perivascular pressure.

Table S1: Slope and mean square error (MSE) of the HD-tangent modulus regression from Figure S3.

Subject	Perivascular Pressure (mmHg)	Slope	Slope Percent Change	MSE
**4-mo ND**	8.22	-6.75E-05	17%	2.09E-06
	13.22	-5.78E-05	0%	1.86E-06
	18.22	-4.96E-05	-14%	2.16E-06
**10-mo ND**	8.22	-8.38E-05	9%	2.57E-06
	13.22	-7.70E-05	0%	2.41E-06
	18.22	-7.41E-05	-4%	2.68E-06
